# “Provoking conversations”: case studies of organizations where Option Grid™ decision aids have become ‘normalized’

**DOI:** 10.1186/s12911-017-0517-2

**Published:** 2017-08-18

**Authors:** Peter Scalia, Glyn Elwyn, Marie-Anne Durand

**Affiliations:** The Dartmouth Institute for Health Policy and Clinical Practice, Dartmouth College, 1 Medical Center Drive 5th floor, Lebanon, NH 03756 USA

**Keywords:** Shared decision making, Option grid™ encounter decision aids, Implementation, Decision support techniques, Normalization process theory, Patient centered care

## Abstract

**Background:**

Implementing patient decision aids in clinic workflow has proven to be a challenge for healthcare organizations and physicians. Our aim was to determine the organizational strategies, motivations, and facilitating factors to the routine implementation of Option Grid™ encounter decision aids at two independent settings.

**Method:**

Case studies conducted by semi-structured interview, using the Normalization Process Theory (NPT) as a framework for thematic analysis. Twenty three interviews with physicians, nurses, hospital staff and stakeholders were conducted at: 1) *CapitalCare* Medical Group in Albany, New York; 2) HealthPartners Clinics in Minneapolis, Minnesota.

**Results:**

‘Coherent’ motivations were guided by financial incentives at CapitalCare, and by a ‘champion’ physician at HealthPartners. Nurses worked ‘collectively’ at both settings and played an important role at sites where successful implementation occurred. Some physicians did not understand the perceived utility of Option Grid™, which led to varying degrees of implementation success across sites. The appraisal work (reflexive monitoring) identified benefits, particularly in terms of information provision. Physicians at both settings, however, were concerned with time pressures and the suitability of the tool for patients with low levels of health literacy.

**Conclusion:**

Although both practice settings illustrated the mechanisms of normalization postulated by the theory, the extent to which Option Grid™ was routinely embedded in clinic workflow varied between sites, and between clinicians. Implementation of new interventions will require attention to an identified rationale (coherence), and to the collective action, cognitive participation, and assessment of value by organizational members of the organization.

**Electronic supplementary material:**

The online version of this article (doi:10.1186/s12911-017-0517-2) contains supplementary material, which is available to authorized users.

## Background

Why are some innovations easier to adopt than others, and what can we learn by examining settings in which innovations are spontaneously adopted? These questions are particularly relevant if we consider patient decision aids. Over 100 randomized trials have shown that their use increases patient knowledge, enhances their understanding of risk and participation in decisions [1]. Nevertheless, it remains difficult to implement these tools in routine practice [2, 3].

Attempts to adopt patient decision aids in routine clinical settings have identified many barriers, and include the healthcare system context, and the various actors, notably, patients, physicians and others in that system [3, 4]. The integration of patient decision aids is difficult, particularly when the tools have to fit into clinical workflows, without increasing time pressures [5, 6]. In addition, the tool’s potential lack of applicability to specific clinical problems and a lack of trust in the information they contain have been cited as significant barriers [6–8].

The International Patient Decision Aids Standards (IPDAS) collaboration has developed a quality framework and criteria for the assessment of patient decision aids [9]. The IPDAS criteria includes health literacy in the development process section, however, very few developers ensure that their tool is usable by patients with low health literacy [9, 10]. Frosch described design challenges: few tools are suitable for minority or disadvantaged groups [11, 12, 13]. Acknowledging and addressing the limitations of these interventions might provide solutions, but there may also be a need to explore what other strategies might exist to support implementation.

Decision aids have a higher likelihood of being adopted if they do not compete with existing ‘priorities, targets and incentives’ [5]. Financial incentives to promote distribution have been shown to have a short term positive effect, but did not lead to sustained use when rewards were withdrawn [14]. Training physicians to use tools and building use into performance feedback can lead to a more sustainable implementation of patient decision aids [15, 16]. Clinical leadership and distributing responsibility across teams are also critical success factors as is organizational investment [17, 18]. Yet, this level of investment is rare [19]. The internal medicine department at the Massachusetts General Hospital in Boston and Dartmouth-Hitchcock Medical Center in New Hampshire are examples of organizations that have made the policy and financial commitment to embed decision aids in clinical systems, but these examples are not commonplace [18].

Given these implementation challenges, an alternative approach to examine successful implementation would be to search for examples where organizations have independently identified and used patient decision aids, where the use of such tools have become embedded in existing workflows independently of research or evaluation efforts.

We had become aware of settings where the Option Grid decision aids for clinical encounters had been adopted or ‘normalized’ as part of routine practice. These tools are one-page, evidence-based summaries of treatment or screening options and their attributes in a tabular format [5, 20]. By using ‘frequently asked questions’, they are designed to promote an informed dialogue between patients and clinicians [21, 22].

To explore and explain the degree to which organizations had embedded the tools into their normal work, we used the Normalization Process Theory (NPT). NPT is a conceptual framework developed to understand the mechanisms that predict the implementation of new interventions into practice settings [23, 24]. NPT has four theoretical tenets: (i) coherence: the ‘sense-making’ that helps people working in the organization reach consensus about the intervention and its aim. In other words, the ability of the members within both organizations to get on the same page through understanding the intervention’s tenets, and the mechanisms underlying its delivery in routine care, realizing how it differs from the existing approach, and the willingness to routinely practice shared decision making in their practice [25]; (ii) collective action: the operational work that allocates tasks to each member of the organization to build and sustain the use of the intervention. The collective action addresses the *how* – how was Option Grid implemented? (iii) cognitive participation: the relational work that influences ‘implementation and legitimation’ of the intervention. In other words, members of an organization working together and organizing themselves to participate in implementing a new intervention; (iv) reflexive monitoring: the communal appraisal work that aids the assessment and comprehension of the effects of the intervention [25]. This means identifying the pros and cons of using Option Grid, and suggesting modifications that would help make the intervention more accessible and easier to integrate into practice settings.

The aim of our study was to use NPT to explore how and why two separate healthcare organizations in the US had spontaneously adopted Option Grid in routine clinical practice, and investigate the factors that have facilitated routine use.

## Methods

A case study design was selected to understand why organizations had decided to use Option Grid routinely, and explore facilitating factors. We chose this design because it enables one to ‘investigate cases in depth and employ multiple sources of evidence where the focus is on a specific situation or context where generalizability is less important’ [26]. The case study design also helped us describe the implementation of the intervention by addressing the how and why [26]. This aligns with our aim to investigate how and why these two organizations implemented Option Grid [26].

### Settings

The sites included in this study had been using Option Grid for over a year before we approached them for their potential inclusion in this case study. We are not aware of, nor did we search for, any other organization who was claiming to have routinely implemented Option Grid in their clinical setting. The case study method does not necessarily require us to include other settings who may have tried, and failed, to implement Option Grid. Case studies focus on the particular in order to provide in-depth assessments. We became aware of the Capital*Care* Medical Group in Albany, because they had inquired about an update to one of the Option Grid decision aids that they had been using. The HealthPartners Practice had informed us of their intention to use Option Grid, which they had identified by their independent search for suitable tools, and because one of the clinicians in this organization had contributed to the development of some of those tools.

Capital*Care* Medical Group in Albany, New York, provides primary care services across a range of practices [27]. The HealthPartners Practice is located at the Riverside Clinic in Minneapolis, Minnesota and at a Specialty Center in neighboring Saint Paul, Minnesota, which provides orthopedic care [28]. Both sites offer a range of medical and dental services, including family practice, internal medicine, pediatrics, Obstetrics, Gynecology, and Nurse Midwifery [27, 28]. We considered our unit of analysis to be the macrosystem (the organization) and the microsystem (the health professionals) who were using the Option Grid decision aids. We interviewed health care providers and staff in each organization who had been familiar with the use of the encounter decision aids. All interviews were conducted by GE and MAD in person at the Capital*Care* and HealthPartners sites. Three group interviews were conducted at Capital*Care.* All interviews were conducted individually with participants at HealthPartners.

### Data collection methods

We conducted semi-structured interviews using a schedule informed by NPT mechanisms (see Additional file 1). Health professionals were asked to: (1) describe their experience with the Option Grid, (2) describe the impact on patients and on their clinical encounters, (3) discuss the impact on the clinic workflow, and to (4) explain whether the tools were helpful in achieving a shared decision making process or not. Interviews and verbal consents were audio-recorded. The study received ethical approval from the Dartmouth College Committee for the Protection of Human Subjects (STUDY00028711).

### Analysis

#### Normalization process theory (NPT)

This theory was developed to investigate the processes by which interventions become part of normal work [23, 24]. The NPT proposes that ‘complex interventions become routinely embedded (implemented and integrated) in their organizational and professional contexts as the result of people working, individually and collectively, to implement them’ [25]. We considered NPT to provide an ideal approach to analyze the study data, and have successfully used this approach before to analyze qualitative data [23–25]. The two organizations did not have rigorous quantitative data, but did list which Option Grid decision aids were used, the number of sites that were using the tools, the total number of times the decision aid was given to patients, and the year of implementation (see Table 1). We were not provided with any other quantitative information regarding the extent to which Option Grid decision aids were used. Therefore, we were not able to investigate how often Option Grid was used, by how many physicians or practitioners, with what percent of eligible patients, etc.

**Table 1:** Capital*Care* and HealthPartners Option Grid metric tracking

Setting	Option Grid decision aid	Number of sites using Option Grid decision aids	Total number of times the decision aid was given to patients	Year of implementation
Capital*Care*	High cholesterol	6	887	2013
	Antibiotics for pharyngitis	2	163	2014
	Sciatica	3	80	2013
	Knee pain	1	41	2013
	Prostate-specific antigen (PSA)	1	32	2014
HealthPartners	Dupuytren’s contracture	2	100^a^	2014
	Carpal tunnel syndrome	2	200^a^	2014
	Trigger finger	2	200^a^	2014

^a^This is an estimated number based on information provided during the interview with the ‘champion’ physician at HealthPartners

We applied the four mechanisms coherence, collective action, cognitive participation and reflexive monitoring to examine *why* Option Grid decision aids had been adopted, who used them, how they were implemented in the workflow, and *how they were understood* to be helpful contributions to the work of the clinic. According to NPT, these four mechanisms require collective and continuous sense-making, commitment, effort and appraisal for the intervention to become ‘normalized’ [24].

#### Qualitative data analysis

Initial descriptive codes were generated based on the four NPT mechanisms by PS. Short-phrase labels were assigned to data sections (in-vivo coding) and additional categorical codes were developed to group codes together. Groups were based on codes that supported or refuted the analytical NPT framework. Once the groups of codes were classified among the four NPT mechanisms, we reviewed them to identify four themes that accurately represented the *how* and *why* these tools were being implemented and the facilitators to routine implementation of decision aid tools. Following the analysis of data, triangulation was performed by asking two colleagues who were not involved in the study (PB and MM) to review the codes and themes, to critically appraise the findings, and to offer alternative explanations for the reported outcomes.

## Results

We conducted semi-structured interviews (see Additional file 1) with health professionals who had used the Option Grid or facilitated its use, and were available to take part in an interview. The manager at Capital*Care* selected health professionals who had experience using Option Grid, who would be interviewed by GE and MAD. At HealthPartners, we asked to interview all people who were engaged in using, or coordinating the use of Option Grid decision aids. We then relied on each site to schedule interviews with those health professionals based on their availability and willingness to participate. Nine interviews were conducted jointly by GE and MAD at the Capital*Care* Medical Group, and comprised of three interviews with physicians, and six other interviews, with a nurse, a staff member, a manager, a medical assistant, an administrator and a clinical quality analyst. Fourteen interviews were independently conducted at HealthPartners Practice, in Minneapolis, Minnesota (by MAD, SA, SG), and comprised of three interviews with physicians, six with licensed practice nurses, three with physician assistants, one manager and one with a hand surgery fellow.

### Case study: The Capital*Care* medical group, Albany

This primary care organization is physician-owned and offers services in family practice, internal medicine and pediatrics from 65 physicians across ten sites [27]. Capital*Care* practices chose to utilize a total of 5 Option Grid decision aids. Capital*Care* was able to provide quantitative data about Option Grid decision aid use through the electronic health record from 2013 until July 2015 (time of data collection in Albany). Table 1 ranks the tools from most to least commonly used by the practices, and indicates the number of practices using each Option Grid, the number of tools that have been given out by each practice, and the year of implementation.

### Case study: The HealthPartners practice, Minneapolis

This organization has over 600 physicians [28]. The lead hand surgeon at the HealthPartners Specialty Center had been an editor for the Dupuytren’s disease, Carpal Tunnel syndrome and Trigger Finger Option Grid decision aids. The organization had been using the encounter decision aids for 1 year before we contacted them for inclusion in the study. HealthPartners did not use a metric to track the use of Option Grid decision aids, however, the lead hand surgeon provided the data presented in Table 1 during the interview.

### Thematic analysis

Our analysis led to the identification of four themes - see Table 2.

**Table 2:** Themes underpinning organizational implementation of Option Grids

1) Coherence - Organizational motivation	
2) Collective Action - The interventions viewed as workable solutions	
3) Cognitive Participation - Slow adaptation to new interventions	
4) Reflexive Monitoring - Assessment of benefits and drawbacks	

We describe the four themes identified and assess how we used the NPT mechanisms to interpret the results.

### Theme 1 organizational motivation

Each organization reported that their overall motivation to practice shared decision making had led to the search for, and adoption of encounter decision aids.

### The Capital*Care* medical group, Albany

Capital*Care* was located in a region that was participating in the Comprehensive Primary Care Initiative (CPCI), initiated by the Centers for Medicare and Medicaid Services (CMS). The CPCI was a primary care quality improvement program focused on promoting patient engagement where practices were required to meet stipulated milestones each year in order to obtain financial incentives offered [29]. To meet one of the milestones, Capital*Care* had introduced ‘shared decision making’ into their organization. To operationalize ‘shared decision making’ they had decided to introduce encounter decision aids, and had identified Option Grids as their preferred tool. To qualify for the financial incentives, reports detailing the use of decision aids with patients were required. The use of the tools had been initiated therefore as a means to an end. Due to this sentiment, and the fact that not all physicians were motivated to use the tool, four of the ten Capital*Care* sites in Albany failed to routinely implement Option Grid. One of the organization’s managers recognized the incoherent, top-down approach to how Option Grid implementation came about:

*‘Our central business office, they kind of pushed us in that direction’* (Site Manager, The Capital*Care* Medical Group, Albany)

*‘Yeah, all we heard from CMS was… go use these tools’ (Site Manager, The* Capital*Care Medical Group, Albany)*

### The HealthPartners practice, Minneapolis

At HealthPartners the use of Option Grid decision aids had been initiated by a hand surgeon, reported in the interviews to have been the primary advocate. The hand surgeon had also played a significant role in the development of the three Option Grid decision aids that were being used in the hand surgery clinic. Data confirmed the importance of this ‘champion’ role as an example for her colleagues:

*‘We just started giving them out in her clinics and then some of the other providers got interested in it and we started rolling it out in their clinics’* (Nurse, The HealthPartners Practice, Minneapolis).

*‘Dr. [champion physician] presented it to the group and encouraged us to try it…she said these [Option Grid] were a great tool, and she thought that we should start to use them’* (Physician, The HealthPartners Practice, Minneapolis)

Coherence: *the ‘sense-making that promotes or inhibits the coherence of the intervention.’*

When we consider these two settings using the ‘coherence’ lens of NPT, we realize that the motivation at the Capital*Care* organization, and to some degree, the consensus for adopting the use of Option Grid, had been stimulated by an external initiative. Capital*Care* wanted to benefit from the financial incentives offered by the CPCI initiative. This ‘sense-making’ management plan was the extrinsic motivation that led to the use of the encounter patient decision aids. In contrast, at HealthPartners, the motivation was intrinsic, in that a ‘champion’ physician had influenced her colleagues by demonstrating the benefit of using the encounter decision aids.

### Theme 2 the interventions viewed as *workable* solutions

The brief nature of the Option Grid decision aid was a workable solution to health professionals at CapitalCare who were looking to satisfy financial incentives set by the organization. The collective action of the nurses at both settings was reported as a key factor in promoting adoption of the tools.

### The Capital*Care* medical group, Albany

Providers had searched for free, seemingly easy-to-use tools that would satisfy the financial incentives set by management, yet not burden the clinical workflow. Using the Option Grid encounter decision aid enabled physicians to enter codes in the patient’s electronic health record, which fulfilled the CPCI requirements for compensation. Some physicians altered the content of the tool yet kept the one-page format:

‘*We had taken the tool, and adjusted it to a way that it would be more patient friendly, more patient literate by changing some of the words and the layout’ (Physician, The CapitalCare Medical Group Albany)*

Others did not understand the difference between shared decision making and patient education, and saw the tool as an information leaflet to be given to patients rather than as a tool to support deliberation about options and to elicit patient preferences:

*‘It’s still a challenge to get them [other clinicians] to understand the difference between shared decision making and patient education’* (Staff Member*,* The Capital*Care* Medical Group, Albany)

*‘I think this is more patient education. So, I see them as an extra educational tool to use.’ (Physician, The CapitalCare Medical Group Albany)*

Capital*Care,* as part of the CPCI program, also asked nurses to identify patients that could benefit by receiving an encounter decision aid, and alert physicians to its possible utility in their clinical encounters:

*‘A lot of our offices are engaged in pre-visit planning. So during that process if the person ... identifies that the patient may be a good candidate for shared decision making, then that individual may flag the record or attach it [the Option Grid] to the encounter form ...’* (Staff Member*,* The Capital*Care* Medical Group, Albany)

### The HealthPartners practice, Minneapolis

Nurses at HealthPartners helped embed the encounter decision aids without much disruption to the existing clinical workflow. Nurses determined patient eligibility for one of the three Option Grid decision aids (Dupuytren’s disease, carpal tunnel syndrome and trigger finger), based on the referral and reported symptoms. Before the surgeon entered the examination room, the nurses reported facilitating a discussion with the patient using the Option Grid content. This method ensured patients were already familiar with the content and comparisons described in the encounter tools ahead of their discussions with the hand surgeons:

*‘So, we, the rooming nurses, give the patients the Option Grid. So, when they’re waiting for the provider to come in the room they can start reading about trigger finger, carpal tunnel ... I think we have a Dupuytren’s one’* (Nurse, HealthPartners Practice, Minneapolis)

Nurses at HealthPartners would also explain to newer staff how best to integrate the tool in the clinic workflow:

*‘That’s a clinical training thing, you know. The more seasoned nurses would be explaining to the newer people who rotate’* (Nurse, HealthPartners Practice, Minneapolis)

All those interviewed emphasized the importance of the nurses as facilitating the adoption of Option Grids, a process made easier in this setting because of the limited number of pre-specified problems:

*‘Well, I talk about all their symptoms and I say, well I feel like you may have carpal tunnel. I have this Grid; it talks about all your treatment options’* (Nurse, HealthPartners Practice, Minneapolis)

Collective Action: *the operational work that enables the enactment of the intervention.*

The brief nature of the Option Grid represented a workable solution for health professionals at CapitalCare to, not only satisfy the financial incentives, but also to modify the content of the tool before providing it to patients. The work performed by nurses at both settings was important to routine adoption of Option Grid. Pre-visit planning by nurses enabled them to attain the appropriate tool for the patient prior to the consultation, thereby alleviating the responsibility to operationalize the tool from the physician. In summary, at both settings, we saw evidence of collective action to adopt the tools by a range of professionals with different roles in the organizations.

### Theme 3 slow adaptation to new interventions

The clinics reported a lack of support and resources, coupled with an absence of formal training as a reason for the challenges initially experienced in adopting the Option Grid decision aids. This was particularly true for the primary care setting at Capital*Care*, where the physicians reported resistance to using the tools.

### The Capital*Care* medical group, Albany

Meetings, tutorials and webinars had been offered to clinicians at Capital*Care*, but a number of physicians interviewed reported a lack of specific training about how to use Option Grids:

*‘I don’t think there’s been any formal training. I mean-no, not really’* (Physician, The Capital*Care* Medical Group, Albany)

*‘A lot of emails, but no formal training’* (Physician, The Capital*Care* Medical Group, Albany)

This lack of training led to both resistance and skepticism, by both staff and physicians:

*‘They [the clinicians] didn’t understand what the benefit was’* (Staff Member, The Capital*Care* Medical Group, Albany)

At the time of our interviews, six out of the ten Capital*Care* clinics had adopted the encounter decision aids in their clinics. Four clinics did not wish to use the tools and the management reported mixed views about the tools:

*‘And there are certain sites that have those physician champions ... and some physicians struggle with the concept, and that’s really where we’re at’* (Site Manager, The CapitalCare Medical Group, Albany)

### The HealthPartners practice, Minneapolis

Interviewees reported initial levels of reluctance to the implementation of the encounter decision aids. Before being introduced to these tools, most surgeons had not been aware of shared decision making, had not used encounter decision aids, nor come across the idea of using these tools collaboratively with patients.

*‘We did active listening in residency and got critiqued on it, but we never actually used a tool’* (Physician, HealthPartners Practice, Minneapolis)

A nurse described this reluctance as a reaction to having to change established practice:

*‘I don’t know if that’s because they don’t like it [the Option Grid], but it’s a different way to practice and they’re used to doing it their way’* (Nurse, HealthPartners Practice, Minneapolis)

Interviewees also reported concerns about time pressure and a lack of perceived utility for some patients as a significant contribution to their hesitation. Physicians reported being selective in their use of the Option Grid, making judgments based on patient characteristics:

*‘So, it’s patient-dependent, obviously. We don’t bring one in every visit’* (Nurse, HealthPartners Practice, Minneapolis)

*‘Well, when we use it, it’s highly variable based on the patient’* (Physician, HealthPartners Practice, Minneapolis)

Cognitive Participation: *the relational work that influences ‘enrolment and legitimation’ of the intervention.*

As these data illustrate, the health care professionals at Captal*Care* and HealthPartners assessed the utility of the tools as members of a work setting, and did so in relation to each other’s reactions and the reactions of patients. Our analysis reveals that over time they gradually saw the usefulness of the tool in their work. On the other hand, their lack of awareness that these tools had been purposefully designed to facilitate shared decision making in clinical encounters led to mixed views about their utility. This was particularly noticeable at Capital*Care* where physician resistance was more vocal, and less of a barrier at HealthPartners because of the way in which the nurses had facilitated the integration of the tools into the clinical workflow.

### Theme 4 assessment of benefits and drawbacks

Participants in both settings reported perceived benefits of using Option Grid decision aids. They also suggested modifications that would help make the interventions more accessible and easier to integrate into practice settings.

### The Capital*Care* medical group, Albany

Participants reported that the encounter decision aids provided benefits, particularly in terms of information provision and as a way for preparing patients:

*‘The importance of your tools, though, is that [they have] evidence-based accurate information’* (Staff Member, The CapitalCare Medical Group, Albany)

*‘It [the Option Grid] provides a structured format for the patient to understand the nature of their health disease and provide information that helps make a better informed decision’* (Physician, The CapitalCare Medical Group, Albany)

Physicians reported improved interactions when the tools were used, and noticed how patients asked more questions and were more satisfied.

*‘I think the people with the grid maybe ask a few more questions. Maybe the grid, for them brings a few things up that they may not have thought of’* (Physician, The CapitalCare Medical Group, Albany)

In addition, a few physicians reported the benefits of using the tools as a means to generate and document different kinds of dialogue with patients:

*‘It’s a great way, in our medical record, to document the conversations we’ve had with patients, the way they’ve changed, the way they’ve thought over the months, and so that’s where I like it’* (Physician, The CapitalCare Medical Group, Albany)

Physicians at Capital*Care* were concerned about the high literacy levels that would be required by patients to make good use of the Option Grid:

*‘We see people ... who have a low literacy rate, so I just want the communication to go both ways’* (Physician, The CapitalCare Medical Group, Albany)

Physicians at Capital*Care* recognized that extra time was needed but were able to adopt the tools by using them with selected patients:

*‘It slowed down the day a little bit, so we had to pick and choose where we were going to use it’* (Physician, The CapitalCare Medical Group, Albany)

### The HealthPartners practice, Minneapolis

Physicians at HealthPartners appreciated patients having access to the evidence-based information presented in the Option Grid. The concise format served to empower patients and improve collaboration:

*‘I think that the quality of the conversation is much better’* (Nurse, HealthPartners Practice, Minneapolis)

*‘I think it is a nice way to just march people through their options and have something on paper that they can see and read, which is nice’* (Physician, HealthPartners Practice, Minneapolis)

*‘Also it makes them feel a bit empowered in that they know what their options are and can pick and choose what they think is best for them’* (Physician, HealthPartners Practice, Minneapolis)

Physicians at HealthPartners raised similar concerns about readability for patients with low health literacy:

*‘The only question of possible concern is some of our patients may be illiterate or have poor health literacy’* (Physician, HealthPartners Practice, Minneapolis)

In contrast to Capital*Care*, interviewees at HealthPartners indicated that using the Option Grid shortened their clinical encounters by ensuring that patients were better informed and better prepared to make decisions:

*‘If anything, slightly shorter [encounters] because the patient has had a chance to read it and go through it, and then they focus in on the area that they like’* (Physician, HealthPartners Practice, Minneapolis)

Reflexive Monitoring: *the appraisal work that aids the assessment of the effects of the intervention.*

Health professionals and managers at both settings had spent time reflecting about the overall contribution of Option Grids to their practice. Some were critical, even skeptical at times, but overall, the data indicates that the organizations as a whole felt that the tools added value rather than brought extra burden.

## Discussion

This case study suggests that patient decision aids that are specifically designed for use in clinical encounters can be embedded in clinical settings, provided there is agreement about the *need* to use them, that the team members are willing to work together to make sure that such tools can be integrated in existing work patterns, and understood as making a positive overall contribution to the work that has to be performed. These considerations match the mechanisms of the NPT, which provides an explanatory framework for understanding the sustained use of these tools by the two systems examined. The motivation for the use of the Option Grid at Capital*Care* was their wish to achieve success in an external quality improvement initiative. At HealthPartners, implementation efforts were motivated by a ‘champion’ physician. The nursing staff also played a pivotal role by systematically identifying eligible patients and providing those patients with the relevant encounter tool. These organizations, in different ways and to different degrees, exhibited *coherence*, *collective action* and *cognitive participation* that supported the sustained use of the tools. The organizational appraisal, in other words, their *reflexive monitoring*, was positive overall, despite concerns about readability and time pressures.

A strength of this study was the in-depth interviews with multiple stakeholders (physicians, nurses, staff members) at two independent clinical service settings. Another strength of this study is the case study method to investigate the two real-world settings who had adopted Option Grid decision aids independent of any invitation. This provides insight into how real clinical practices address the issue of patient decision aid implementation.

We recognize that two study authors (GE and MA) are involved in the Option Grid Collaborative, a non-profit making organization that develops and disseminates patient decision aids for clinical encounters. To mitigate the risk of bias, the analysis was conducted by PS. The addition of other settings would have given us access to data from organizations that tried to implement Option Grid in their practice, but did not succeed, or organizations that chose not to adopt Option Grid. We were not privy to any quantitative data regarding how often Option Grid decision aids were used, by how many clinicians, with what percentage of eligible patients, or any demographic information regarding the patients (including patient health literacy levels). This would have strengthened the study findings, which we recognize.

Implementing patient decision aids into clinical settings is a difficult process [1, 4, 25, 30]. In the UK, an implementation program known as MAking Good Decisions In Collaboration (MAGIC) highlighted the need for an organizational *coherence*, i.e. a widely held and agreed understanding of SDM principles in order to facilitate the implementation of patient decision aids [25]. Commitment at multiple organizational levels has been recognized as an important precondition for implementation [1, 4, 31]. This lack of commitment was noticeable at the Capital*Care* sites that did not use patient encounter tools. A systematic review identified that a key implementation success factor is the presence of a respected clinical champion [1]. This was reinforced by the MAGIC program when it demonstrated the power of clinical leaders to recruit colleagues [25].

Whether the introduction of incentives is a sustainable way to encourage the implementation of patient decision aids is a matter of debate [32]. Management decisions at Capital*Care* were clearly made as a response to financial incentives but this interest did not translate into consistent motivation among the front-line clinicians. In contrast, at the HealthPartners setting, the willingness to adopt the encounter tools on the basis of their perceived inherent value to both clinicians and patients; financial incentives were not instrumental. These two contrasting strategies can be used as examples by other organizations looking to implement decision aids. This study indicates that financial incentives may not be the best approach for implementing decision aids in comparison to using a ‘champion’ clinician that can lead by example.

Few studies have examined the implementation of patient decision aids in routine service systems, and this case study work needs replication in many other settings. More work is needed to understand how social context and the relationships between actors within the social system affects ‘real world’ implementation [33]. There is evidence emerging that encounter tools are effective: when physiotherapists were trained how to use an Option Grid developed for patients with osteoarthritis of the knee, shared decision levels and patient knowledge increased, without an increase in encounter duration [22]. Examination of videotapes showed that using encounter tools led to parents asking more questions and getting more involved in the clinical discussion about circumcision [34]. Encounter tools seem to be viewed as ‘flexible artifacts’, allowing physicians to adapt how they use the tools, making it easier to embed the interventions into clinical work [31, 35].

Future studies should also consider ideas to support the implementation of patient decision aids. Organizations may want to consider providing clinicians with shared decision making workshops to facilitate the approach and clarify any questions or concerns they may feel about using a decision aid. To ease the time pressures on clinicians, organizations can also enable patients to use an interactive tool via their electronic health record before the encounter, so they can bring their results to the clinician with any questions they may have. If organizations choose to implement a financial incentive to promote shared decision making, then it may be worthwhile to invest in modifying the electronic health record to make it easier for health professionals to indicate the use of decision aids to satisfy the financial incentives. This can help bypass administrative barriers that may impede the success of implementation. In the future, moving decision aids to an online platform can also lead to the creation of algorithms to customize decision aids for each individual patient according to their profile and health literacy.

## Conclusion

The data in these two case studies illustrate the gap between research and practice. Research has shown the effectiveness of patient decision aids, including those that are designed for use in clinical encounters. However, providing proof of effectiveness is not enough to ensure that practice settings will adopt these interventions. The tools have to successfully pass the tests of ‘normalization’ - an agreed rationale for using them, agreements to work together to make the implementation successful, and agreement that the benefits outweigh the burden of change and new processes. These two case studies provide some evidence that Option Grid decision aids are meeting some of these requirements, although reservations were also expressed. Implementation of new tools and practices will require methods to assess whether they can successfully fulfill the requirements of ‘normalization’ and to examine, what, if anything, could facilitate a normalization process.

## Additional file

Additional file 1: Interview guide. (PDF 689 kb)

## Abbreviations

CMS: Centers for Medicare and Medicaid Services
CPCI: Comprehensive Primary Care Initiative
MAGIC: MAking Good Decisions In Collaboration
NPT: Normalization Process Theory
