# Supplementary material for: “Provoking conversations”: case studies of organizations where Option Grid™ decision aids have become ‘normalized’
Source: BMC Med Inform Decis Mak. 2017 Aug 18;17:124. doi: 10.1186/s12911-017-0517-2 (PMC5562992; doi:10.1186/s12911-017-0517-2)
Supplement: Additional file 1: — Interview guide. (PDF 689 kb) [file 12911_2017_517_MOESM1_ESM.pdf]

Appendix 1.

1) INTERVIEW GUIDE – OTHER STAFF's PERSPECTIVES

Using Option Grid in Routine Clinical Settings

A. Experience with Option Grid decision aids to date

We understand that MDs in your clinic have been using Option Grid decision aids for a little while.

1. ESTABLISHED USE: Could you tell me a little more about how Option Grids are used in this clinic?

Probe: First, could you specify your role in the Clinic?
Probe: Who uses Option Grids? How often?
Probe: Which Option Grid(s) are they using?
Probe: How long have they been using it for?
Probe: Are MDs using the Grids with all clinically eligible patients? How do they choose which patients to use it with?
Probe: How are Option Grids typically used? How are they introduced to patients? By who?
Probe: Do some MDs refuse to use them? Why?
Probe: Probe: How does it compare to a consultation without the Option Grid?

2. EARLY USE: Thinking back about when Option Grids were introduced …
Probe: Can you describe how the use of the Option Grid was initiated? Who had the idea? What was your reaction?
Probe: How were Option Grids introduced to MDs?
Probe: What were their reactions? What did the MDs think of the Option Grids?
Probe: Were they prepared to use it?
Probe: Was training offered? If not, do you think it was needed?
Probe: How did Option Grids become part of your clinic's usual practice?
Probe: What were, if any, the key challenges to routine use? How were they overcome?
Probe: Did it become easier over time? Why?
Probe: Has anything changed since using the Option Grid for the first time xx months ago? Are the Option Grids being used or introduced to patients differently since initial use?

3. Why are you still using Option Grids now?

Probes: Are there, for you, any clear benefits to using it? Or are you using it for other reasons? Benefits to patients?

Probe: Could you explain what those benefits are?

4. What have you found most difficult or challenging about using Option Grids?

Probe: How could this be improved?

B. Perceived Fit in Clinic Workflow and Routine Care

5. Do Option Grids fit into the clinic workflow?

Probe: If so, how?
Probe: Did you have to make changes to the clinic flow in order to use the Option Grids?
Probe: What factors in your office made it easy to use? Where any other changes needed?
Probe: If yes, why?
Probe: Is there any evaluation?
Probe: Is it part of 'what is done' (normalized)?
Probe: Is there any plan for endorsing/embedding Option Grids in the clinic? Or do you consider that this has already happened?
Probe: Is there any plan for embedding Option Grids in exiting systems? Could it be accessible via the Electronic Health Record?
Probe: Do you think these tools need to be marketed directly to patients?

6. Why would other clinicians want to use Option Grid routinely?

Probe: Are tools like Option Grids considered helpful or problematic by MDs?
Probe: Based on your experience so far, can you anticipate barriers to widespread use?
Probe: Do you think that using Option Grids requires training? How?
Probe: Would you recommend it to other clinics and colleagues?
Probe: Could it be used by other members of the medical team?

C. Implementing Shared Decision Making

10. Forgetting about Option Grids for a moment, how else have you promoted shared decision making in the clinic?

Probe: Have you used other decision aids? Why? How do these compare to Option Grids?
Probe: Have you received training in shared decision making? Is there a need for it?
Probe: What is the best way of engaging patients and families in decision making about all aspects of care?
Probe: What are the main barriers?

11. Do you have any other comments about the implementation of shared decision making in your clinic?

12. Do you have any other comments about using Option Grids in your clinic?

We would like to thank you very much for your time.

2) INTERVIEW GUIDE – CHIEF MEDICAL OFFICER'S PERSPECTIVE

Using Option Grids in Routine Clinical Settings

A. Experience with Option Grids to date

We understand that health care professionals in your clinics have been using Option Grids since 2013.

1. Could you tell us a little more about how this happened?
Probe: Why did you decide to promote and implement shared decision making?
Probe: How did you first come across Option Grids?
Probe: Why did you decide to use Option Grids?
Probe: Did you consider other alternatives?

2. What do you think about Option Grids?
Probe: How about the content? Format? Layout?
Probe: What could we change? Improve?
Probe: How do you think they are currently being used?

2. EARLY USE: Thinking back about when Option Grids were introduced …
Probe: Can you describe how the use of the Option Grid was initiated? Who had the idea?
Probe: How were Option Grids introduced to staff in your Clinics?
Probe: What were their reactions?
Probe: Was training offered?
Probe: Did Option Grids become part of the clinics' usual practice? If so, how?
Probe: What were, if any, the key challenges to routine use? How were they overcome?

3. Why are you still using Option Grids now?

Probes: Benefits to health care professionals? Benefits to patients? Time? Cost? Other?
Probe: Could you explain what those benefits are?

B. Perceived Fit in Clinic Workflow and Routine Care

4. Do Option Grids fit into the clinics' workflow?

Probe: If so, how?
Probe: Did you have to make changes to the clinic flow in order to use the Option Grids?
Probe: Is there any evaluation?
Probe: Is it part of 'what is done' (normalized)?
Probe: Is there any plan for endorsing/embedding Option Grids in the clinic? Or do you consider that this has already happened?
Probe: Is there any plan for embedding Option Grids in exiting systems? Could it be accessible via the Electronic Health Record?
Probe: Do you think these tools need to be marketed directly to patients?

5. Why would other clinicians/other health care systems want to use Option Grid routinely?

Probe: Are tools like Option Grids considered helpful or problematic?
Probe: Based on your experience so far, can you anticipate barriers to widespread use?
Probe: Do you think that using Option Grids requires training? How?
Probe: Would you recommend it to other clinics and colleagues?

C. Implementing Shared Decision Making

6. Forgetting about Option Grids for a moment, how else have you promoted shared decision making in the clinic?

Probe: Have you used other decision aids? Why? How do these compare to Option Grids?
Probe: Have you trained staff in shared decision making? Is there a need for it?
Probe: What is the best way of engaging patients and families in decision making about all aspects of care?
Probe: What are the main barriers?

11. Do you have any other comments about the implementation of shared decision making in clinic?

12. Do you have any other comments about using Option Grids or other shared decision making interventions?

We would like to thank you very much for your time.

3) INTERVIEW GUIDE – HEALTH CARE PROFESSIONALS' PERSPECTIVES

(for HCP who are using the Option Grids with patients)

Using Option Grids in Routine Clinical Settings

A. Experience with Option Grids to date

We understand that you have been using Option Grids in your clinic for some time.

1. ESTABLISHED USE: Could you tell me a little more about your experience of using Option Grids so far?

Probe: How long have you been using it for? How often do you use it? with all clinically eligible patients? If not, why not? How do you decide whether or not to use it with a patient?
Probe: Which Option Grid(s) are you using?

Probe: How do you find those Grids? Difficult/easy to use?
Probe: How do you use them? How do you normally introduce it to patients? Do you give it to them or do they typically bring it to the visit?
Probe: Can you estimate how long it takes to use it?
Probe: How does it compare to a consultation without the tool?
Probe: How are your colleagues finding it?
Probe: Could you estimate how many of your colleagues are using the tool(s) now?

2. EARLY USE: Thinking back about when you first started using Option Grids (e.g. first and second use)…

Probe: Can you describe how the use of the Option Grid was initiated? Who had the idea? What did you think?
Probe: Why did you first start using it?
Probe: Did you feel comfortable using the Option Grid? If not, what about it felt awkward?
Probe: What were your colleagues' reactions?
Probe: Did you receive any training/information as to how best to use it with patients?
Probe: Did it become easier to use over time?
Probe: Has anything changed since using the Option Grid for the first time xx months ago? Are you using the tool differently now, compared to your first attempt?
Probe: Are patients responding differently compared to early use?

3. Why are you still using Option Grids now?

Probes: Are there, for you, any clear benefits to using it? Or are you using it for other reasons? Benefits to patients?
Probe: Could you explain what those reasons/benefits are?

4. What have you found most difficult or challenging about using Option Grids?

Probe: How could this be improved?
Probe: What do you think about its content?
Probe: Did you have to adapt it to individual patients? How?
Probe: Was it easy for patients to understand?

B. Perceived Impact on Consultation and on Patients

5. Has it changed the conversation you are having with patients?

Probe: How? Can you give an example?
Probe: What has been the impact, if any, on the consultation?
Probe: Has it lengthened the consultation? Has it made the consultation more focused and efficient?
Probe: What is the impact on your patients? Do patients behave differently compared to consultations without the Option Grid? How?
Probe: What are the benefits for patients? What are the drawbacks?

Probe: Are patients generally willing to give their opinion and participate in the decision making process?
Probe: Do patients typically take it home?
Probe: How do families/caregivers respond to it?

6. How does using the Option Grid affect the decision making process?

Probe: Does it facilitate or hinder the decision making process?
Probe: Does it help patients make an informed decision? How?
Probe: Are patients ready to make a treatment decision after using the Option Grid? More prepared than without the Option Grid?
Probe: Is the decision generally made in the consultation?
Probe: Have you noticed any differences in patients' decision making process compared to consultations where the tool wasn't used?

C. Perceived Fit in Clinic Workflow and Routine Care

7. Do Option Grids fit in the current workflow?

Probe: If so, how?
Probe: Did you have to make changes to the clinic flow in order to use the Option Grids?
Probe: What factors in your office made it easy to use?
Probe: If yes, why?
Probe: Is there any plan for endorsing/embedding Option Grids in the clinic? Or do you consider that this has already happened?
Probe: Is there any plan for embedding Option Grids in exiting systems? Could it be accessible via the Electronic Health Record?
Probe: Do you think these tools need to be marketed directly to patients?

8. Why would other clinicians want to use Option Grid routinely?

Probe: Are tools like Option Grids considered helpful or problematic by clinicians?
Probe: Based on your experience so far, can you anticipate barriers to widespread use?
Probe: Do you think that using Option Grids requires training? Why? How?
Probe: Would you recommend it to your colleagues ?
Probe: Could it be used by other members of the medical team?

D. Implementing Shared Decision Making

9. Forgetting about Option Grids for a second, how else have you promoted shared decision making with your patients?

Probe: Have you used other decision aids? Why? How do these compare to Option Grids?
Probe: Have you received training in shared decision making? Is there a need for it?
Probe: What is the best way of engaging patients and families in decision making about all aspects of care?

Probe: What are the main barriers?

10. Do you have any other comments about the implementation of shared decision making in your clinic?

11. Do you have any other comments about using Option Grids, or anything that particularly struck you when using it?

We would like to thank you very much for your time.
